# A novel impact-tip crusher catheter enhances mechanical lithotripsy for large common bile duct stones

**DOI:** 10.1055/a-2630-2208

**Published:** 2025-07-15

**Authors:** Akihiro Maruyama, Makoto Kobayashi, Ryota Tagawa, Junya Yamada, Hiroki Kato, Shintaro Tominaga

**Affiliations:** 1 37036 Department of Gastroenterology, Yokkaichi Municipal Hospital, Yokkaichi, Mie, Japan; 2 36590 Department of Gastroenterology, Nagoya University, Nagoya, Aichi, Japan

Mechanical lithotripsy is commonly used for large common bile duct (CBD) stones; however, fragmentation can be difficult, especially in cases of hard or large stones 1 2 3 . We evaluated the utility of a novel crusher catheter, Stone Smash (Boston Scientific, Marlborough, MA, USA), which is equipped with a distal impact tip that doubles as a basket guide ( Fig. 1 ). This device was jointly developed by our institution and Lake R&D Co., Ltd. (Okaya, Nagano, Japan). Conventional basket catheters apply circumferential compression to the entire stone, leading to a crushing pattern in which the stone is flattened and fractured 4 . In contrast, the impact tip on Stone Smash enables direct, longitudinal force application at a single point, causing the stone to split along its vertical axis. Additionally, to improve ductal insertion, the sheath is short and flexible, with a preshaped bendable tip that allows easy advancement even without a guidewire. We conducted a bench test comparing Stone Smash and a standard crusher catheter without an impact tip. Five calcium sulfate blocks of similar size were used for each device, and the average crushing force required for fragmentation was measured. Stone Smash required a mean force of 5.6 kgf, significantly less than the 8.2 kgf needed with the conventional catheter ( *p* < 0.05). We performed lithotripsy using the Stone Smash in a 78-year-old man with CBD stones measuring 22 and 14 mm. The procedure was completed successfully without any complications ( Video 1 ). This novel catheter demonstrated superior crushing force compared to standard devices and provided excellent maneuverability due to its flexible sheath and short-type guidewire compatibility. These features enabled smooth bile duct insertion, even in tortuous anatomy or in the absence of a guidewire. Stone Smash appears to be a valuable tool in the endoscopic management of difficult CBD stones.

**Fig. 1:**
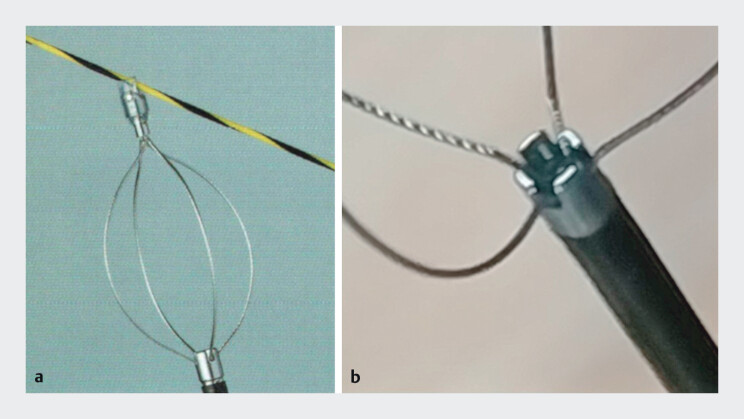
**a** Basket with high expansion force prevents stone escape. **b** Claw-equipped metallic tip allows effective and concentrated lithotripsy.

Demonstration of the Stone Smash catheter used for endoscopic lithotripsy of a common bile duct stone. The catheter is inserted into the bile duct over a guidewire or directly without it. After capturing the stone within the basket, the gradual closure of the claw-equipped metallic tip enables effective fragmentation.Video 1

Endoscopy_UCTN_Code_TTT_1AR_2AH

## Notes

Endoscopy E-Videos https://eref.thieme.de/e-videos
*E-Videos* is an open access online section of the journal *Endoscopy* , reporting on interesting cases and new techniques in gastroenterological endoscopy. All papers include a high-quality video and are published with a Creative Commons CC-BY license. Endoscopy E-Videos qualify for HINARI discounts and waivers and eligibility is automatically checked during the submission process. We grant 100% waivers to articles whose corresponding authors are based in Group A countries and 50% waivers to those who are based in Group B countries as classified by Research4Life (see: https://www.research4life.org/access/eligibility/ ). This section has its own submission website at https://mc.manuscriptcentral.com/e-videos .
